# Properties of Sn-Doped PBZT Ferroelectric Ceramics Sintered by Hot-Pressing Method

**DOI:** 10.3390/ma17205072

**Published:** 2024-10-17

**Authors:** Dagmara Brzezińska, Dariusz Bochenek, Maciej Zubko, Przemysław Niemiec, Izabela Matuła

**Affiliations:** Institute of Materials Engineering, Faculty of Science and Technology, University of Silesia in Katowice, 75 Pułku Piechoty 1 A, 41-500 Chorzów, Poland; maciej.zubko@us.edu.pl (M.Z.); przemyslaw.niemiec@us.edu.pl (P.N.); izabela.matula@us.edu.pl (I.M.)

**Keywords:** PBZT ceramics, relaxors, ferroelectrics, phase transition, dielectric properties

## Abstract

This work investigated the structure, microstructure, and ferroelectric and dielectric behavior of (Pb_0.97_Ba_0.03_)(Zr_0.98_Ti_0.02_)_1−*x*_Sn*_x_*O_3_ (PBZT_*x*Sn) solid solution with variable tin content in the range *x* = 0.00–0.08. Synthesis was carried out using the powder calcination method, and sintering was carried out using the hot-pressing method. For all the PBZT_*x*Sn samples at room temperature, X-ray diffractograms confirmed the presence of an orthorhombic (OR) crystal structure with space group P*nnm*, and the microstructure is characterized by densely packed and properly shaped grains with an average size of 1.36 µm to 1.73 µm. At room temperature, PBZT_*x*Sn materials have low permittivity values *ε*′ ranging from 265 to 275, whereas, at the ferroelectric–paraelectric phase transition temperature (RE–C), the permittivity is high (from 8923 to 12,141). The increase in the tin dopant in PBZT_*x*Sn lowers permittivity and dielectric loss and changes the scope of occurrence of phase transitions. The occurring dispersion of the dielectric constant and dielectric loss at low frequencies, related to the Maxwell–Wagner behavior, decreases with increasing tin content in the composition of PBZT_*x*Sn. Temperature studies of the dielectric and ferroelectric properties revealed anomalies related to the phase transitions occurring in the PBZT_*x*Sn material. With increasing temperature in PBZT_*x*Sn, phase transitions occur from orthorhombic (OR) to rhombohedral (RE) and cubic (C). The cooling cycle shifts the temperatures of the phase transitions towards lower temperatures. The test results were confirmed by XRD Rietveld analysis at different temperatures. The beneficial dielectric and ferroelectric properties suggest that the PBZT_*x*Sn materials are suitable for micromechatronic applications as pulse capacitors or actuator elements.

## 1. Introduction

Piezoelectrics are materials that can realize the mutual conversion of mechanical and electrical energy and vice versa [1,2]. For many decades, the PbZr_1−*u*_Ti*_u_*O_3_ (PZT) solid solution has been widely used in many fields of modern technology and microelectronics due to its favorable dielectric, ferroelectric, piezoelectric, and pyroelectric properties [3,4,5].

The general formula of the PZT solid solution is (*u*)PbTiO_3_-(1 − *u*)PbZrO_3_, in which changing the Zr/Ti ratio and the use of appropriate doping make it possible to obtain a wide range of functional properties of electroceramics for specific applications [6,7]. At room temperature, with high zirconium contents (*u* = 0.05–0.53), PZT has a rhombohedral structure (RE), and, with high titanium contents (*u* = 0.47–0.95), PZT has a tetragonal structure (T). At the same time, for *u* = 0.00–0.05, PZT exhibits an orthorhombic structure (OR). In the paraelectric state (above the transition temperature), PZT has a cubic structure (C). In the tetragonal phase, there are 180° and 90° domains. Reorientations of the 180° domains due to an external electric field or mechanical stress do not cause stresses, while reorientations of the 90° domains result in the appearance of elastic deformations near the domain boundaries. In turn, in the rhombohedral phase, there are 180° and 71°/(109°) domains, and the crystal structure can occur in a low-temperature RE(II) or high-temperature RE(I) variety. The low-temperature RE(II) phase is characterized by both shifts in the Pb(Zr/Ti) atoms in the (111) direction as well as rotations of the oxygen octahedra around this direction. When the RE(II) phase transitions to the high-temperature RE(I) phase at *T* = 100 °C, there is only a system of polar shifts of atoms up to a temperature of 250 °C. In the PZT compositions belonging to this area, there are two forms of order, i.e., electrical (with polar shifts) and orientational (with deformation of the unit cell) [8]. In the two-phase system of rhombohedral and tetragonal phases (for *u* approximately 0.48), the number of different types of domain walls increases, increasing the possibilities of domain reorientation under external factors. This leads to an increase in the piezoelectric parameters, permittivity, and polarization values in this area, which are particularly interesting in application [3,9]. Such advanced PZT-type materials with functional properties are widely used in various fields, such as mechanical engineering, robotics, automotive, medical and biomedical, aviation, electronic terminals, national defense science and technology, microelectronics, and micromechatronics [10,11,12]. Especially, the PZT-type materials with high piezoelectricity are commonly used in many devices, such as various sensors, precision actuators, ultrasonic transducers, capacitors, memories, devices for mechanical energy harvesters, and microwave, SAW, and smart devices [11,13,14].

The increased application possibilities are achieved by designing multicomponent compounds based on PZT (due to doping the basic PZT composition), improving their functional properties, and increasing the stability of their parameters. Thanks to this solution, highly efficient ferro-piezo materials are created [11]. The wide isomorphism of PZT enables substituting both pentavalent and trivalent ions in the Zr/Ti positions, which act as donors and acceptors, respectively [9]. Donor 5+ dopants, due to the reduction in the number of oxygen vacancies, increase the volume resistance of the ceramics. On the other hand, acceptor 3+ dopants reduce the grain resistance, causing a slowdown in the movement of domains due to increased oxygen vacancies [15]. In PZT ceramic materials, oxygen vacancies cannot be avoided due to the evaporation of lead during the high temperatures of the sintering process [16]. Many works have been published so far on modifying the main composition of PZT with various dopants, by substituting both the Pb and Zr/Ti positions [17,18,19]. For example, doping with higher-valence donor ions instead of Pb (e.g., Sm^3+^, La^3+^, Bi^3+^, and Nd^3+^) or in the Zr/Ti positions (e.g., Nb^5+^, Sb^5+^, and W^6+^) leads to the formation of lead vacancies, promoting domain deviation in the electric field. This results in an increase in the residual polarization *P*_r_ and a decrease in the coercive field *E*_c_ [20,21]. The introduction of lower-valence acceptor ions instead of Pb (K^+^ and Na^+^) or in the Zr/Ti positions (e.g., Fe^2+^, Fe^3+^, Co^2+^, Co^3+^, Mn^2+^, Mn^3+^, Ni^2+^, Cr^3+^, and Mg^2+^) generates oxygen vacancies and the so-called pinning effect, as a result of which the ceramics will show an increased coercive field but reduced residual polarization [20,21,22]. Kelley et al. observed a significant enhancement in the electromechanical response in BaTiO_3_ induced by the controlled injection of oxygen vacancies [23]. Tai et al. showed that, in BiFeO_3_−BaTiO_3_ material, the increasing oxygen vacancies enhance the transient piezoelectric response. However, the aging behavior deteriorates with increasing oxygen vacancy concentration, leading to the degradation of the piezoelectric performance [24]. In the case of industrial production, obtaining repeatable properties of ceramic materials in the technological process is extremely difficult, among other aspects, due to their high hardness and brittleness, difficulties in obtaining the stoichiometry of the composition resulting from low forming efficiency, complex technological processes, and disadvantages regarding the traditional obtaining methods [25]. PZT-type materials are mainly obtained by the free sintering method (FS, pressureless) due to its simplicity and low cost [26,27]. Other sintering methods used include, for example, microwave sintering [26,28], spark plasma sintering (SPS) [19,29,30,31,32], flash sintering [33], the hot-pressing method (HP) [19,29], and the cold sintering process (CSP) [34,35]. The development of nanotechnology in recent years has forced the need to control the grain growth during the sintering of nanostructured PZT [36]. Several methods have been developed for the synthesis of one-dimensional nanostructured PZT, such as hydrothermal, ultrasonic spray combustion synthesis, electrohydrodynamic atomization, sol–gel routs, co-precipitation, and electrospinning [9,36,37,38]. Recently, new methods for obtaining PZT-type materials such as Ink-Jet Printing [39], the stereolithography method [40], 3D printing [41], and vat photopolymerization (VPP) in the additive manufacturing (AM) process have been proposed, which have created new possibilities in the preparation of advanced ceramics with high precision and surface quality [25].

Also, introducing an isovalent dopant to the mainly PZT composition causes changes in the electrophysical properties of the solid solution. For example, introducing a Ba^2+^ dopant into the lead position increases the so-called ferroelectric hardness. Handerek et al. in [42] studied the dielectric properties of the ceramic (Pb_0.75_Ba_0.25_)(Zr_0.70_Ti_0.30_)O_3_ (PBZT), which is a solid solution of the rhombohedral ferroelectric PBZ and the tetragonal ferroelectric PBT. In the studied composition, they revealed typical relaxor behavior, in which the dielectric constant value decreased and the maximum shifted towards higher temperatures with increasing frequency. For example, the attractive properties of PBZT crystals at low titanium contents were presented in [43]. It was shown that even a low PbTiO_3_ content introduced to the PbZrO_3_ firmly changes and provides new properties to the newly formed solid solution. Ti ions induce the formation of polar regions even in the paraelectric phase [44] and significantly extend the temperature range of intermediate-phase existence [45]. It has been shown that both point defects and Ti ions can influence the range and nature of the intermediate phase (ferroelectric or a mixture of paraelectric, ferroelectric, and antiferroelectric phases) in PZT material [43]. The inspiration for researching the PBZT ceramic material was interesting literature reports on the unique properties of crystals with similar compositions, including structural change occurring near the ferroelectric and antiferroelectric phase boundary [43]. Also, there are no literature reports on the study of PBZT material doped with tin and sintered by hot pressing, which is an aspect of novelty in this work. In this work, the (Pb_0.97_Ba_0.03_)(Zr_0.98_Ti_0.02_)_1−*x*_Sn*_x_*O_3_ (PBZT_*x*Sn) tin-doped material for *x* = 0.00–0.08 was obtained using the HP method, and X-ray, microstructural, dielectric, and ferroelectric tests and XRD Rietveld analysis at different temperatures were subsequently carried out.

## 2. Materials and Methods

### 2.1. Material and Technological Process

The research material was a solid solution of the PbZr_0.98_Ti_0.02_O_3_ type modified with Ba barium and Sn tin. In the basic composition, barium was substituted for lead (position A of the compound), and, in all compositions, it had a constant amount (*y* = 0.03). In turn, tin was substituted in the B position of the compound (in place of Zr/Ti) with a variable amount from the range *x* = 0.00–0.08. The general chemical formula of the tested material was as follows: (Pb_1−*y*_Ba*_y_*)(Zr_0.98_Ti_0.02_)_1−*x*_Sn*_x_*O_3_ (PBZT_*x*Sn). In the technological process, the synthesis of the PBZT_*x*Sn material was carried out using the powder calcination method. In contrast, the sintering of the compacts was carried out using the hot-pressing method. The following reagents in technology were used, i.e., BaCO_3_ (99.99%, POCH, Gliwice, Poland), TiO_2_ (99.9%, Merck, Darmstadt, Germany), ZrO_2_ (99.5%, Aldrich, St. Louis, MO, USA), PbO (99.9%, POCH, Gliwice, Poland), and SnO_2_ (99.9%, Aldrich, St. Louis, MO, USA). The powders were weighed according to the stoichiometric ratio with an excess of PbO (5%) and then mixed with ethanol for 15 h at 250 rpm rotating speed of the planetary mill (FRITSCH Pulverisette 6, Idar-Oberstein, Germany). Then, a mixture of powders was calcined at *T_calc_* = 850 °C/*t_calc_* = 3 h and ground again after the calcination process. Next, synthesized powders of PBZT_*x*Sn material were compacted on a hydraulic press under a pressure of 300 MPa (samples with dimensions of 10 mm in diameter and 3 mm in thickness). Sintering was carried out by hot pressing under *T_s_* = 1200 °C/*t_s_* = 2 h/*p_s_* = 20 MPa. Five compositions of (Pb_0.97_Ba_0.03_)(Zr_0.98_Ti_0.02_)_1−*x*_Sn*_x_*O_3_ (PBZT_*x*Sn) materials were obtained for *x* = 0.00–0.08, which have been marked as PBZT_0Sn (for *x* = 0.00), PBZT_2Sn (for *x* = 0.02), PBZT_4Sn (for *x* = 0.04), PBZT_6Sn (for *x* = 0.06), and PBZT_8Sn (for *x* = 0.08). After the sintering process, the ceramic samples were ground and polished to a thickness of 1 mm. In order to remove mechanical stresses that occurred during grinding, the samples were annealed at conditions of 750 °C/15 min. The PBZT_*x*Sn ceramic samples were prepared for dielectric tests by applying silver paste to both surfaces of the samples by firing method at a temperature of 850 °C/15 min.

### 2.2. Characterization

The phase composition of the material obtained was studied by X-ray diffraction (XRD) with a Philips X’Pert PW 3040/60 diffractometer (PANalytical, in Almelo, the Netherlands) equipped with a copper anode lamp (CuK*α λ* = 1.54178 Ǻ) and a graphite monochromator. The patterns were recorded by “step-scanning” with 0.04 degrees within the 2*θ* angle range from 5° to 145° and a 40 kV acceleration voltage at room temperature (RT). The line profile standard for instrumental broadening was NIST standard SRM660a (LaB6 powder). The analysis was performed using HighScore Plus software version 3.0d (3.0.4, produced by PANalytical B.V., Almelo, The Netherlands) and the ICDD PDF-5+ database. The temperature-dependent X-ray diffraction measurements were performed with a Malvern Panalytical (Malvern Instruments, Malvern, UK) Empyrean diffractometer using a nickel-filtered Cu K_α1,2_ radiation (*λ*  =  1.5406 Å) and equipped with a PIXcell^3D^ ultra-fast solid-state hybrid detector. The diffractometer was also equipped with an Anton Paar (Anton Paar, Graz, Austria) TTK 450 temperature chamber. Measurements were carried out at the selected temperatures of 30 °C, 90 °C, 130 °C, 170 °C, and 230 °C during heating cycle and 170 °C, 130 °C, 90 °C, and 60 °C during cooling cycle. The measurements were performed in a reflection mode, in the Bragg–Brentano geometry (*θ*–*θ* scan technique), within the 2*θ* range of 10–90°. The Rietveld refinement was performed using the FullProf program suite [46].

The relative density of PBZT_*x*Sn ceramic samples was estimated using the Archimedes method. The sample surfaces were sputtered with gold for microstructural tests using Smart Coater DII-29030SCTR (Jeol, Ltd., Tokyo, Japan). The analysis of energy dispersive spectrometry (EDS) and microstructures of fracture ceramic samples (SEM) were performed using Jeol Field Emission Scanning Electron Microscope (JSM-7100 TTL LV, Jeol Ltd., Tokyo, Japan). ImageJ software (ImageJ 1.37v, LOCI, University of Wisconsin-Madison, WI, USA) was used to calculate the average grain size using SEM microstructural images. The dielectric measurements were measured during the heating cycle using the QuadTech LCR meter (QuadTech, Maynard, MA, USA) in the range 20 to 400 °C and at frequencies from 500 Hz to 1.0 MHz. Hysteresis loops *P–E* were tested using a high-voltage amplifier (Matsusada Precision Inc., HEOPS-5B6, Kusatsu, Japan) and a Sawyer-Tower circuit [47] at RT. A National Instruments NI USB-6002 digital card was used to capture the data (National Instruments, Austin, TX, USA), while the control program of the measuring system was written in the LabView environment.

## 3. Results and Discussion

### 3.1. Structural Test

**Figure 1:**
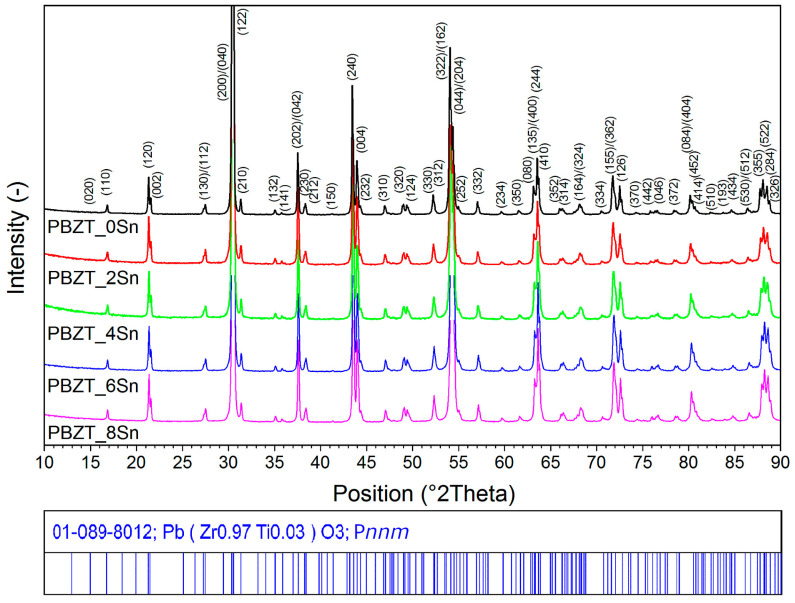
XRD patterns of the PBZT_*x*Sn ceramic powders at room temperature.

The X-ray diffraction patterns of the PBZT_*x*Sn ceramic powders are presented in Figure 1. For all the samples, the analysis revealed the presence of reflections belonging to the main composition of Pb(Zr_0.97_Ti_0.03_)O_3_ (01-089-8012 ICDD PDF-5+) with an orthorhombic crystal system with space group P*nnm* and lattice parameters *a* = 5.8629 Å, *b* = 11.7401 Å, and *c* = 8.2069 Å [48]. In the studied material, distinct peaks of doublets belonging to orthorhombic phases (202)/(042) and (230)/(212) were recorded for angles 2*θ* equal to 37.65° and 38.4°, respectively. In the crystal structure, Zr atoms displace along the z-axis in the crystal structure, and local disorder occurs in the oxygen substructure [48]. Figure S1 (Supplementary Materials) shows two enlarged diagram fragments of the X-ray diffraction patterns. In the angle region 2*θ* = 42°–45° (Figure S1a), a shift in the doublet orthorhombic phase (240)/(004) is observed towards higher angles for the composition with increasing amounts of Sn. Similarly, in the region of angles 2*θ* = 53°–55° (Figure S1b), the increase in tin causes a shift in the (322)/(162)/(044)/(204) peaks. It may indicate a decreased distance between the ions in the B positions of the unit cell with increased Sn in PBZT. It is due to the difference in the size of the ionic radii of the replaced ions (*r*_Zr_ = 0.72 Å) and the substitution ions (*r*_Sn_ = 0.69 Å) introduced into the B positions of the compound [49]. According to [50], the substitution of Sn^4+^ in Pb(Zr,Ti)O_3_ into the B (Zr, Ti)^4+^ position of the compound reduces the distortion of the ferroelectric phase unit cell, decreasing the volume difference between the ferroelectric and antiferroelectric phases.

The average crystallite size was determined based on the measurements, and Rietveld analysis was performed using FullProf software version 7.95 (Jan2023-ILL JRC). The analysis was performed based on the broadening diffraction lines compared to the apparatus profile function. The silicon standard sample was measured before the measurements, and the diffraction line parameters were refined. Based on the results obtained using the full pattern calculation, the average crystal size was determined to be 1182(3) Å. The X-ray diffraction method provides an average size of the coherent diffracting domains, usually much smaller than the overall grain size. Grain boundaries can contain disordered regions, defects, and voids that do not contribute to coherent X-ray diffraction, meaning that the crystallite size is measured only within individual coherence domains.

### 3.2. Microstructure

**Figure 2:**
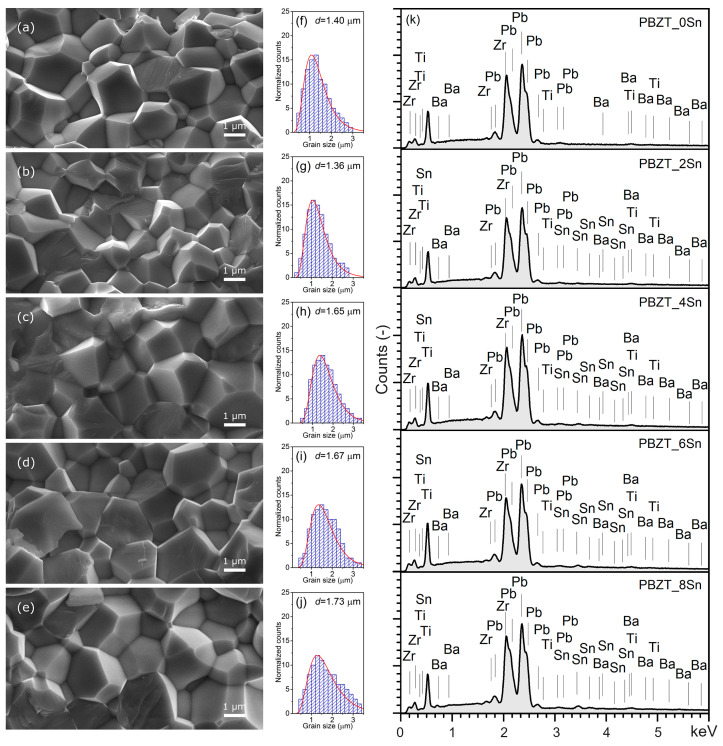
Microstructures and statistical results of the grain size distribution of PBZT_*x*Sn ceramic samples obtained using HP technology: PBZT_0Sn (**a**,**f**), PBZT_2Sn (**b**,**g**), PBZT_4Sn (**c**,**h**), PBZT_6Sn (**d**,**i**), PBZT_8Sn (**e**,**j**); (**k**) EDS analysis image of the element distribution for the PBZT_*x*Sn ceramic samples.

Figure 2 shows microstructural SEM images of the PBZT_*x*Sn ceramic samples. The SEM images show densely packed and properly shaped grains with strongly outlined grain boundaries. The fracture occurs mainly at the grain boundaries, which indicates the ceramic samples’ high mechanical strength and confirms the technological process’s correctly selected parameters. The average grain size for the PBZT_*x*Sn material ranges from 1.36 µm to 1.73 µm. The PBZT_2Sn composition has the most excellent uniformity of grain size and the smallest average grain size (1.36 µm) (Figure 2b). The largest grains are characterized by the composition with the highest tin content (PBZT_8Sn), with an average grain size of 1.73 µm (Figure 2e). In [51], ceramic samples of the material (Pb_0.95_Ba_0.05_)(Zr_1−*u*_Ti*_u_*)O_3_ prepared by the solid-state reaction method were obtained, which showed a fine-grained microstructure. However, these samples were characterized by improperly shaped grains, and the microstructure was characterized by high grain size inhomogeneity. In [52], the adopted technological conditions had a positive effect on the microstructure of the material (Pb_0.925_Ba_0.075_)(Zr_1−*u*_Ti*_u_*)O_3_, showing more greater correctness of grain shape but with significantly larger grains compared to our results. The obtained PBZT_*x*Sn ceramic materials exhibit high material density (from 7.34 to 7.60 g/cm^3^). However, earlier studies presented in [53] have shown that using the SPS sintering method (while maintaining optimal sintering conditions) enables obtaining a fine-grained microstructure and increasing the density of the PBZT_*x*Sn material. Thus, considering the influence of the applied sintering method on the density of ceramic samples, the most advantageous sintering method of the PBZT_*x*Sn material seems to be SPS. At the same time, the least satisfactory results are obtained in the classical sintering method [54]. The ceramic samples of the PBZT_*x*Sn material sintered by hot pressing have intermediate density values.

Figure 2k shows the results of the surface EDS analysis for the PBZT_*x*Sn material, while Table 1 summarizes the results of the experimental studies and theoretical calculations for the individual compositions. EDS analysis was performed on a larger area of the ample surface, and the experimental results are the average result of five randomly selected areas of the surface microstructure of the ceramic sample. The qualitative EDS analysis showed the presence of all the elements assumed in the designed chemical compositions and the absence of foreign elements and impurities. The quantitative EDS analysis revealed a slight excess of lead oxide and deficiencies of ZrO_2_, TiO_2_, SnO_2_, and BaO compared to the theoretical amounts. It may be due to the excess PbO used during the technological process of using the PBZT_*x*Sn material.

### 3.3. Ferroelectric Properties

**Figure 3:**
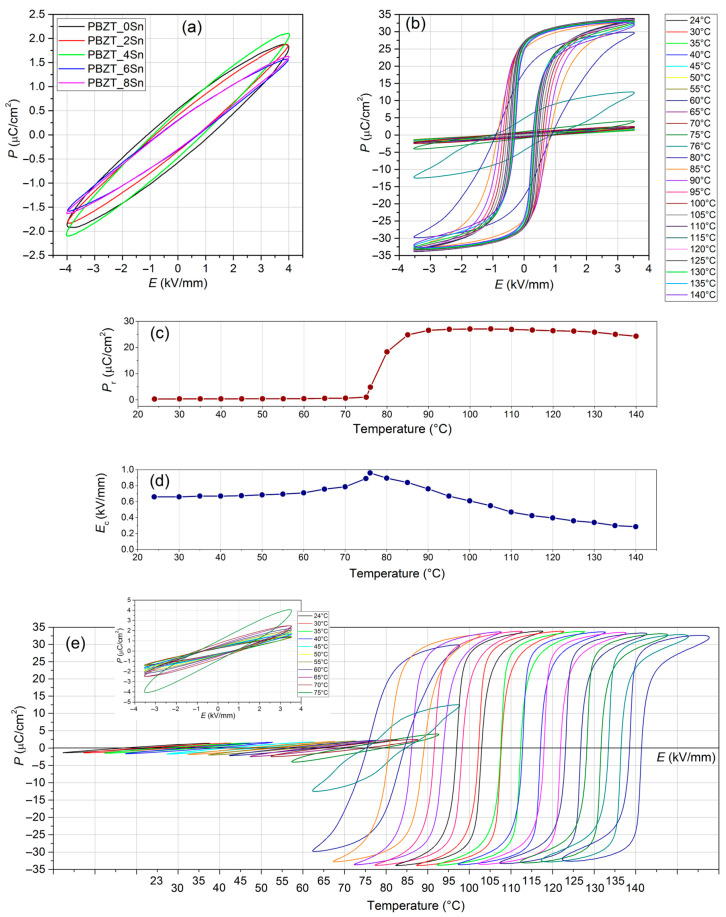
(**a**) *P*–*E* hysteresis loops for PBZT_*x*Sn ceramic samples at room temperature and *E* = 4 kV/mm, (**b**) temperature *P–E* loop for PBZT_6Sn sample, (**c**) temperature dependence of remnant polarization and (**d**) coercive field, and (**e**) temperature *P*–*E* loop for PBZT_6Sn sample with shift in results by a constant value along the OX axis.

Figure 3a shows the ferroelectric properties of the PBZT_*x*Sn ceramics (for a field of 4 kV/mm) in the form of a ferroelectric *P*–*E* hysteresis loop. At room temperature, the PBZT_*x*Sn material exhibits an orthorhombic phase (OR), which results in low values of residual polarization *P*_r_ and low saturation of the *P*–*E* loop. The residual polarization values range from 0.30 μC/cm^2^ to 0.53 μC/cm^2^, while the maximum polarization values *P*_m_ range from 1.52 μC/cm^2^ to 2.08 μC/cm^2^ (Table 2). At room temperature, the samples exhibit a reasonably wide hysteresis loop (coercive field *E*_c_ = 1.14 kV/mm for PBZT_0Sn), which narrows with the increase in tin in PBZT (*E*_c_ = 0.69 kV/mm for PBZT_8Sn). The increase in temperature causes a structural transformation (OR–RE) in the PBZT_*x*Sn material. In ferroelectric studies, this is manifested in a rapid increase in polarization (above 75 °C) and obtaining an intense saturation of the hysteresis loop (above 90 °C). Figure 3b shows the change in the *P*–*E* hysteresis loop with increasing temperature (performed every 5 °C) for an exemplary PBZT_6Sn composition in the temperature range from RT to 140 °C. At room temperature, the ceramics exhibit low values of residual polarization (0.26 μC/cm^2^) and maximum polarization (1.38 μC/cm^2^), while, above the observed OR–RE transition, the *P*_r_ and *P*_m_ polarization values increase to 26.74 μC/cm^2^ and 33.62 μC/cm^2^, respectively (Figure 3c). Up to a temperature of 130 °C, the values of the above parameters remain at a similarly high level. The coercive field values *E*_c_ as a function of temperature exhibit slightly different behavior (Figure 3d). With the increase in temperature, an increase in the coercive field value is observed (at RT *E*_c_ = 0.66 kV/mm), while, at a temperature of approximately 76 °C, a maximum of the *E*_c_ value occurs, which is 0.96 kV/mm. After exceeding it, a slight decrease in the coercive field value is observed (Figure 3d). A good presentation of the obtained results and the changes in the ferroelectric properties of the tested material in the vicinity of the OR–RE transformation region can be obtained by the compilation of the *P*–*E* hysteresis loops created every 5 °C on one graph with the *P*–*E* graphs shifted by a constant shift value on the OX axis (Figure 3e). As a supplement, within Figure 3d, the collective *P*–*E* plots obtained below the OR–RE transformation temperature are also included, which show the width of the hysteresis loop of a series of samples in the orthorhombic OR phase. Comparing the results of the temperature studies of the ferroelectric hysteresis loop for the PBZT_6Sn sample sintered with different technologies (i.e., hot pressing in the present work and classical method in [53]), it can be stated that, in the classical method, the initiation of the OR–RE structural transformation occurs at a higher temperature (the rhombohedral phase appears at approximately 90 °C). Hysteresis loops have higher values of coercive field (~1.46 kV/mm), but the values of residual polarization (20.6 μC/cm^2^) and maximum polarization (23.8 μC/cm^2^) in the rhombohedral phase RE are much lower [53]. Figure 3c,d show that the temperature changes in the residual polarization *P*_r_, the maximum polarization *P*_m_, and the coercive field *E*_c_ in two adjacent phases (orthorhombic and rhombohedral) occur relatively stably. The rapid changes in the *P*_r_ and *E*_c_ parameters occurring in a wide temperature range between these phases indicate the coexistence of both phases in this area.

The ferroelectric ceramics’ coercive field depends on the microstructure’s grain size [55]. Large grains can have a well-developed domain structure, in which the movement of domain walls will take place at a relatively low electric field, and, consequently, the coercive field decreases with the increase in grain size. However, when Pb is in the PZT composition, the excess PbO can cause stress inside the grain and distortion of the lattice. The resulting stress effectively blocks the movement and alignment of the domain, thus necessitating a larger external electric field for domain reorientation [55]. Generally, in ferroelectric materials, the increase in temperature affects the movement of domain walls, but, in the orthorhombic phase, the changes in polarization and coercive field are small. As shown in Figure 3c,d, pronounced changes in parameters *P*_r_ and *E*_c_ occur only when the ferroelectric phase appears. In the wide area of coexistence of two orthorhombic and rhombohedral phases, there is an increase in the polarization *P*_r_ and a maximum value of coercive field *E*_c_. Above 115 °C, *P*_r_ decreases due to the so-called pyroelectric effect [56]. In the temperature range of the rhombohedral phase, with increasing temperature, the *E*_c_ value decreases due to the decrease in the interfacial energy of the ferroelectric domains, which makes the movement of the domain walls easier. The temperature-dependent change in the shape of the hysteresis loop indicates the movement of the domain walls, crystal structure, and internal spontaneous reorientation of polarization [56]. The changes observed in a wide area indicate the coexistence of two phases and the phase transition from orthorhombic to rhombohedral. Similar phenomena have been observed in several previous works in PZT-based materials with high Zr content [57] and in PMN-PT-type solid solutions [56].

### 3.4. Dielectric Properties

**Figure 4:**
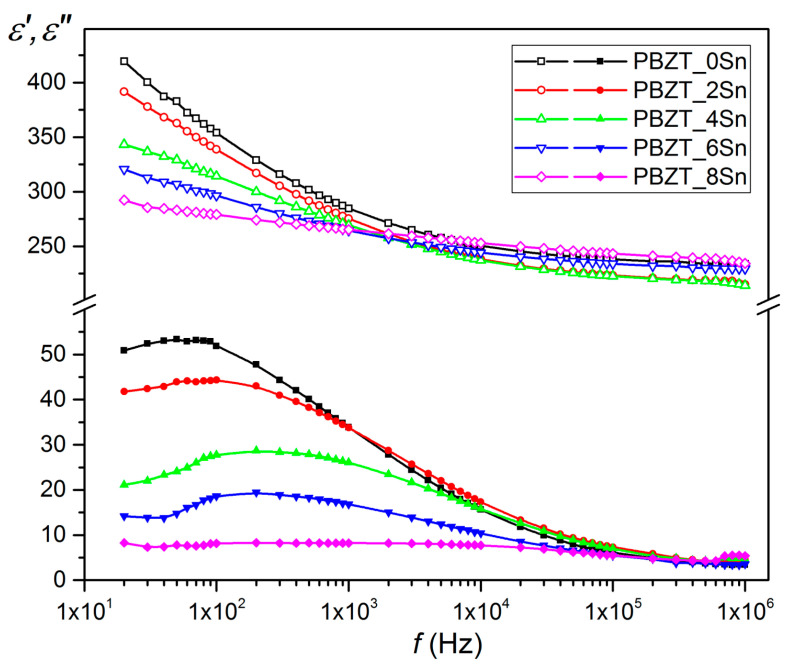
The variation in the dielectric permittivity as a function of frequency for PBZT_*x*Sn ceramic samples.

Figure 4 shows the changes in the real part of the dielectric constant *ε*′ and dielectric loss *ε*′′ for the PBZT_*x*Sn material as a function of frequency in the range from 20 Hz to 1 MHz. The highest values of the dielectric constant are found in the undoped material (composition PBZT_0Sn), which also has the highest values of dielectric loss. At room temperature, the dielectric constant and dielectric loss exhibit dispersion at low frequencies (Figure 4), which decreases with increasing tin content in the PBZT_*x*Sn composition. At lower frequencies, both *ɛ*′ and *ɛ*″ have large values that decrease with increasing frequency. The observed phenomenon in the ferroelectric materials can be explained based on the Maxwell–Wagner two-layers model of polarization [58] for space charge or interfacial polarization and Koop’s phenomenological theory [59]. This model describes dielectric materials as a system consisting of fairly conductive grains separated by grain boundaries with higher resistance [60]. In this case, under the influence of an applied external field, electrons (*e*^−^) can easily migrate from the interior of the grains and accumulate at the grain boundaries [61]. Thanks to this, the material obtains a high dielectric constant and polarization. However, the increased frequency of the applied field causes the electrons to reverse their motion, reducing the probability of reaching the grain boundary and decreasing polarization [61]. This low-frequency behavior suggests that conduction mechanisms play an essential role in the dielectric response of ceramic materials [62]. In the *ε*′′ (*T*) graphs, there is a strongly diffuse relaxation peak, the shape of which results from the superposition of low-frequency conduction. The observed maximum occurs at lower frequencies than in [63].

**Figure 5:**
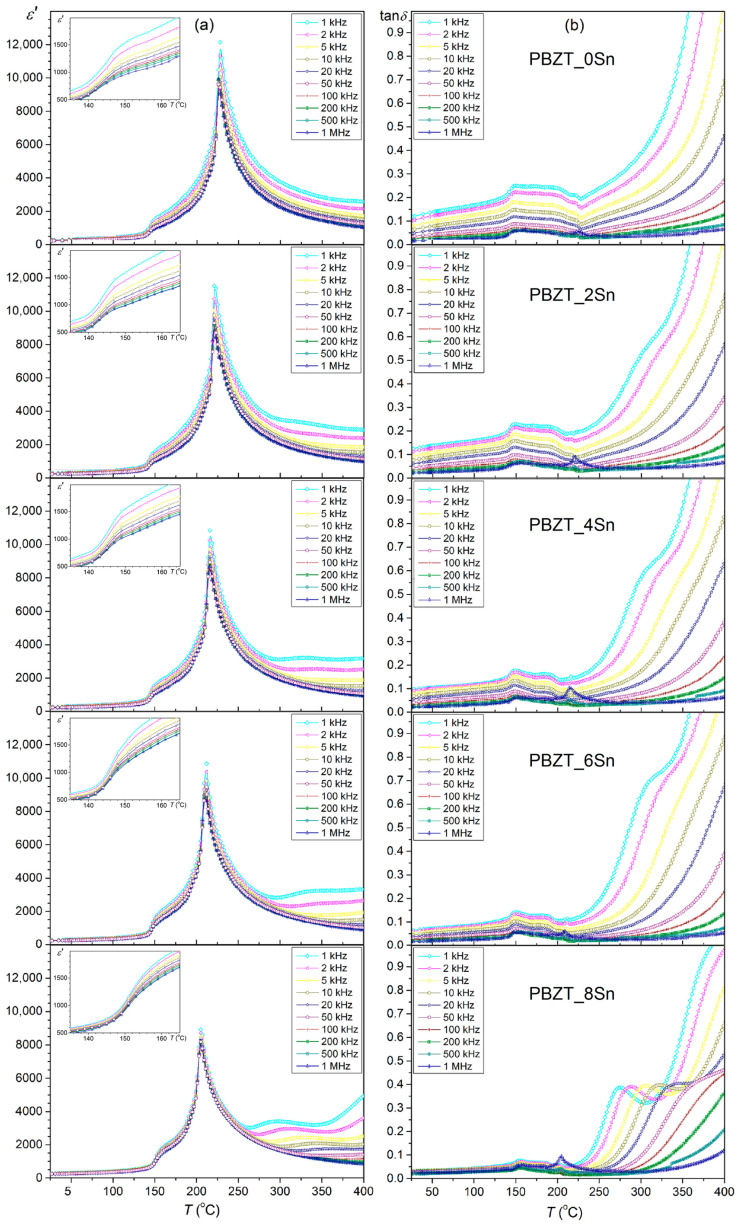
Permittivity (**a**) and dielectric tangent loss (**b**) vs. temperature of PBZT_*x*Sn ceramics.

Figure 5a presents the temperature studies of the permittivity for the PBZT_*x*Sn material. At room temperature, all the samples have low permittivity values ranging from 265 to 275 (Table 2), and, with increasing temperature, the permittivity increases significantly. In the temperature graphs *ɛ*(*T*) for the PBZT_*x*Sn material, several characteristic phenomena can be observed, which occur in different temperature regions. The first anomaly occurs in the temperature range from 148 °C to 155 °C and is manifested by a rapid increase in permittivity. The observed anomaly is related to the thermal and structural transformation from the orthorhombic OR phase to the rhombohedral RE phase. The second phenomenon observed in the temperature range from 205 °C to 228 °C, with a distinct sharp maximum of permittivity at *T*_m_, corresponds to the ferroelectric–paraelectric phase transition. The PBZT_*x*Sn material exhibits high permittivity values at the phase transition temperature *T*_m_, which are 12,141, 11,526, 10,850, 10,861, and 8923 for PBZT_0Sn, PBZT_2Sn, PBZT_4Sn, PBZT_6Sn, and PBZT_8Sn, respectively. The trend includes increasing tin content in the PBZT material and decreasing permittivity values *ε*_r_ and *ε*_m_ (Table 2). In [64], for a comparable composition of the PBZT material, the phase transition from the ferroelectric phase to the paraelectric phase occurs in a similar temperature range, showing a sharp phase transition characteristic of PZT-type materials from the morphotropic region. However, the maximum permittivity values are twice as low as the *ε*_m_ values for the material presented in this work. In the *ε*(*T*) graphs above the phase transition temperature *T*_m_, there is also a third anomaly, i.e., dielectric dispersion that is related to a conductivity phenomenon, which is related to the increase in defect mobility (a high-temperature relaxation) [65,66]. Usually, space charge relaxations are related to conductivity phenomena, and the relaxation frequency is corrected by defect occurrence (charge carriers) that depends on the synthesis conditions [66].

It is commonly accepted that dielectric relaxation in oxide materials in the high-temperature region is usually associated with oxygen vacancies, and dielectric relaxation associated with oxygen vacancies, as a rule, occurs in the low-frequency region [64,67,68]. A similar phenomenon of high-temperature relaxation was presented in [64,69], where the results of the temperature dielectric tests of PZT material in higher temperature ranges were presented. The high-temperature dielectric relaxation phenomenon intensifies with increased Sn content in the main PBZT composition (Figure 5a).

The dielectric properties of the PBZT_*x*Sn material are also influenced by the sintering method used in the technological process. The permittivity values for those samples obtained by hot pressing are higher than in the classical method of obtaining ceramics. However, they have slightly lower values than the series of samples obtained by the SPS method [53]. Figure 5b shows temperature-dependent dielectric loss diagrams for a series of PBZT_*x*Sn samples. At room temperature, PBZT_*x*Sn ceramics exhibit low dielectric loss that ranges from 0.031 to 0.123 (Table 2). The dielectric loss decreases as the Sn content in the main PBZT composition increases. Similarly to the temperature measurements of permittivity, the transformations occurring in the PBZT_*x*Sn material are clearly visible on the temperature tan*δ*(*T*) diagrams in the form of various anomalies appearing in different temperature areas. The first anomaly in the form of a local maximum observed in lower temperature ranges (~150 °C) is associated with the occurring OR–FE structural transformation. In turn, in the higher temperature range, i.e., ~200–225 °C, the anomaly that appears is related to the presence of the FE–PE phase transition of the PBZT_*x*Sn material. With further temperature increases, the dielectric loss increases significantly due to increased electrical conductivity. In [64], for a comparable PBZT material composition, similar values of dielectric loss were obtained, but their increase at higher temperatures is rapid.

**Figure 6:**
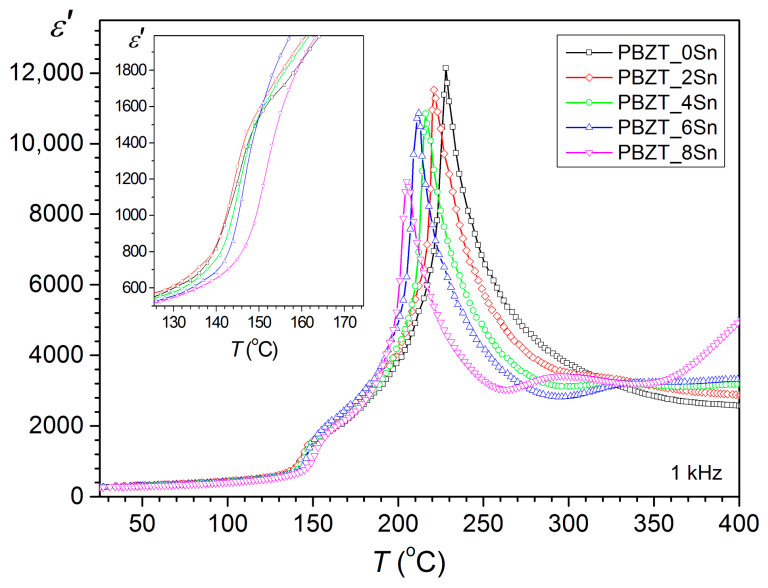
*ε*(*T*) graph for PBZT_*x*Sn material measured at 1 kHz in the heating cycle.

Figure 6 shows a summary graph of permittivity as a function of temperature for a series of tested compositions of the PBZT_*x*Sn material measured at 1 kHz in the heating cycle. The analysis showed the influence of the tin dopant on the dielectric properties and the temperatures of the phase transitions. The increase in the tin dopant in PBZT_*x*Sn shifts the phase transition temperature from the orthorhombic OR phase to the rhombohedral RE phase (the transition occurs at a higher temperature). In the case of ferroelectric–paraelectric phase transition (RE–C), the opposite trend occurs, i.e., the increase in Sn content in the PBZT_*x*Sn material lowers the RE–C transition temperature. A decrease in the maximum value of permittivity *ε*_m_ is also observed at the phase transition point *T*_m_.

**Figure 7:**
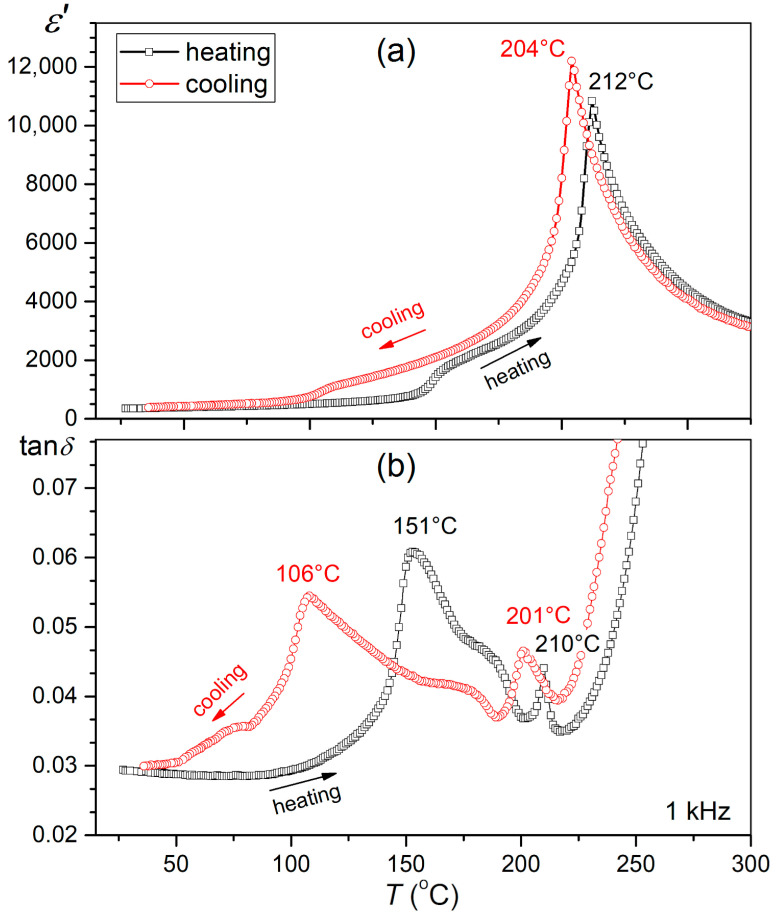
Temperature studies of dielectric properties (**a**) *ε*(*T*) and (**b**) tan*δ*(*T*) for the PBZT_6Sn sample measured at 1 kHz in the heating and cooling cycles.

Figure 7 shows the temperature studies of the dielectric properties of permittivity and dielectric loss angle tangent (in an enlarged range) for the reference sample PBZT_*x*Sn performed for the heating and cooling cycles. Both in the permittivity and dielectric loss graphs (in the heating and cooling cycles), the phase transitions occurring in the material from the orthorhombic phase to the rhombohedral phase (OR–RE) and from the ferroelectric rhombohedral phase to the paraelectric cubic phase (RE–C) were revealed. In the orthorhombic phase, the permittivity remains low, but, after the transition to the rhombohedral phase, the permittivity rapidly increases. The maximum value of electric permittivity occurs at the temperature of the ferroelectric–paraelectric phase transition (*T*_m_), with a sharp character of the phase transition. In the heating cycle, the structural transformation (OR–RE) occurs in the broader temperature range of 140 °C–155 °C, while the RE–C phase transition occurs at *T*_m_ = 212 °C. In the cooling cycle, the studies have shown a shift in the ranges of characteristic structural transformations towards lower temperatures. Thus, the *T*_m_ temperature decreases to 204 °C, and the range of the RE-OR transformation is revealed in the lower temperature range (110 °C–90 °C). Moreover, on the *ε*(*T*) and tan*δ*(*T*) graphs in the cooling cycle, a flattening of the effects of the presence of the OR–RE transformation is observed, which is more strongly outlined in the heating cycle. In the tan*δ*(*T*) graph (Figure 7), the shifts in the characteristic maxima occurring during cooling are also clearly visible. The maximum tan*δ* for the OR–RE transformation for the heating cycle occurs at a temperature of 151 °C and in the cooling cycle at 106 °C. In turn, the characteristic tan*δ* maximum, which usually appears just before the ferroelectric–paraelectric transition (RE–C) for heating, occurs at a temperature of 210 °C and in the cooling cycle at 201 °C. The observed thermal hysteresis in Figure 7 can be explained by the high mobility of ions in the paraelectric phase [70]. Similar anomalies in the temperature profiles of the dielectric properties occurring during the heating and cooling cycles have been presented in several thematic works for the PZT material from the low-titanium-content area, e.g., [43,57,71], and in PMN-PT-type solid solutions [56]. Several previous works have observed similar phenomena in PZT-based materials with high Zr content [57].

### 3.5. Temperature X-ray Tests

**Figure 8:**
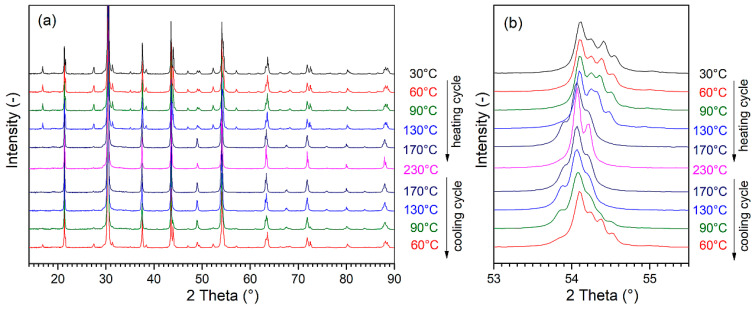
Temperature-dependent XRD patterns of the PBZT_*x*Sn ceramics; (**a**) full measurement range for 2*θ* angles (14°–90°); (**b**) the reflection peaks around 2*θ* = 54°. The beginning of the heating cycle from 30 °C to 230 °C and then the cooling cycle to 60 °C.

**Figure 9:**
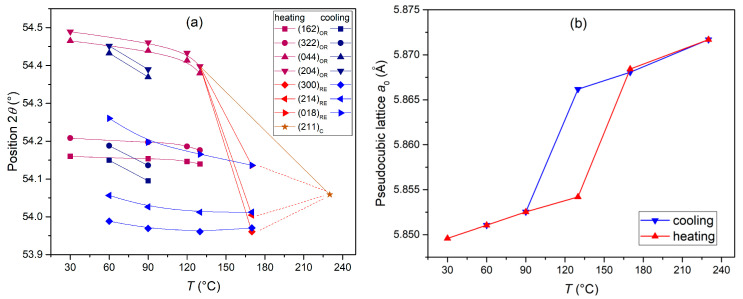
(**a**) The reflection positions of the Rietveld refinements for each XRD pattern of the PBZT_6Sn ceramics and (**b**) the evolution of the pseudo-cubic lattice parameters in the heating and cooling cycles.

In order to confirm the phase transitions revealed by the ferroelectric and dielectric studies, temperature X-ray studies were performed in the observed temperature ranges and heating and cooling cycles (Figure 8). The measurements were performed at several selected temperatures, i.e., 30 °C, 90 °C, 130 °C, 170 °C, and 230 °C. The evolution of the characteristic diffraction reflections belonging to the individual phases is observed in the X-ray images with the change in the measurement temperature. Figure 9a shows a broader measurement range of the measured 2*θ* angles (14°–90°), while Figure 9b shows an enlarged area with 2*θ* angle ranges of 53°–55.5°. At room temperature, the PBZT_*x*Sn material exhibits an orthorhombic structure. During heating, the structural transformation to the rhombohedral phase was revealed in the temperature range from 130 °C to 170 °C. Another phase transition from the rhombohedral ferroelectric phase to the cubic paraelectric phase occurs between 170 °C and 230 °C. The occurring quadruple of the OR phase (322)/(162)/044)/(204) observed at angles 2*θ* = 54.2°–54.5° gradually disappears with temperature and transforms into a triplet of the RE phase (300)/(214)/(018) in the angle range 2*θ* = 54.0°–54.3°, and then into a singlet of the cubic C phase (211) at an angle of 2*θ* = 54.1°. Similarly to dielectric studies, the measurement in the cooling cycle shifts the characteristic ranges of phase transitions towards lower temperatures. During cooling, the rhombohedral phase appeared between temperatures of 230 °C and 170 °C, while the rhomboidal phase began to emerge between 130 °C and 90 °C. At 90 °C, the coexistence of two phases (rhombohedral and orthorhombic) was recorded, which gradually disappears, and, at 60 °C, the orthorhombic phase dominates with trace amounts of the rhombohedral phase. In perovskite materials, the transition from a cubic (paraelectric) system to a ferroelectric system is associated with a change in the volume of the unit cell [57,71]. The fitting parameters performed using the Rietveld method for the PBZT_6Sn sample at individual temperatures for the heating and cooling cycles are listed in Table 3. The X-ray studies that were performed correlate well with the temperature studies of the dielectric parameters. Detailed analysis using the Rietveld method for the cooling cycle measurements also showed that, at 90 °C, the share of the orthorhombic phase in the compound is 21(1)%, while the share of the rhombohedral phase is 79(1)%. At 60 °C, the orthorhombic phase predominates (its percentage share is 62(1)%), while the share of the rhombohedral phase is 38(1)%. Figure 9a shows the positions of the Rietveld enhancement reflections for each XRD pattern, clearly illustrating the occurring phase transitions in the PBZT_*x*Sn ceramics observed during the heating and cooling cycles in the research results presented above. On the other hand, Figure 9b shows the evolution of the pseudo-cubic lattice parameters, which were calculated for all the measured temperature points in the heating and cooling cycles. The visualization of the pseudo-cubic lattice parameters expressed by parameter *a*_0_ of the unit cell also clearly shows the evolution of the pseudo-cubic cell changes and confirms the observed differences during the heating and cooling cycles.

## 4. Conclusions

In this work, the (Pb_0.97_Ba_0.03_)(Zr_0.98_Ti_0.02_)_1−*x*_Sn*_x_*O_3_ (PBZT_*x*Sn) tin-doped material for *x* = 0.00–0.08 was sintered by the hot-pressing method. The X-ray, microstructural, dielectric, and ferroelectric tests and XRD Rietveld analysis at different temperatures were carried out. At room temperature, the PBZT_*x*Sn samples demonstrated an orthorhombic crystal structure with space group P*nnm*. The microstructure of the PBZT_*x*Sn ceramics sintered by the hot-pressing method showed densely packed and correctly shaped grains with strongly outlined grain boundaries. The average grain size ranged from 1.36 µm to 1.73 µm. The microstructural analysis showed no clear trend in the grain size change in the microstructure with increasing amounts of tin in the PBZT_*x*Sn composition. The smallest average grain size (1.36 µm) was demonstrated by the PBZT_2Sn composition and the largest by the PBZT_8Sn composition (1.73 µm). At room temperature, PBZT_*x*Sn materials have low permittivity values in the range of 265 to 275, while, at the phase change temperature, they have high values in the range of 8923 to 12,141. The increase in the tin dopant in PBZT_*x*Sn lowers the permittivity and dielectric loss and changes the ranges of phase transitions. The OR–RE phase transition temperature shifts towards higher temperatures, while the RE–C transition occurs at lower temperatures. At room temperature, the dielectric constant and dielectric loss exhibit dispersion at low frequencies. This phenomenon, called Maxwell–Wagner behavior, decreases with increasing tin content in the PBZT_*x*Sn composition. At room temperature, PBZT_*x*Sn exhibits low values of residual polarization *P*_r_ (in the range from 0.30 μC/cm^2^ to 0.53 μC/cm^2^) and low ferroelectric hysteresis loop *P*–*E* saturation. The structural transformation (OR–RE) occurring in PBZT_*x*Sn increases the residual and maximum polarization and high saturation of the hysteresis loop (above 90 °C for PBZT_6Sn). Detailed temperature studies of the dielectric and ferroelectric properties for the PBZT_6Sn composition revealed anomalies indicating the presence of phase transitions. In the *ε*(*T*) graphs in the heating cycle, the first transformation from the orthorhombic phase to the rhombohedral phase (OR–RE) occurs in the temperature range from 140 °C to 155 °C. In turn, the transformation from the ferroelectric rhombohedral phase to the paraelectric cubic phase (RE–C) occurs at a temperature of (212 °C). The cooling cycle shifts the phase transition temperatures towards lower temperatures. The Rietveld analysis performed based on X-ray measurements at different temperatures confirmed the structural changes occurring in the material. For the example sample PBZT_6Sn, the change in structure from orthorhombic to rhombohedral (OR–RE) was recorded in the temperature range from 130 °C to 170 °C, while, from the rhombohedral phase to the regular (RE–C) phase, the change was between temperatures of 170 °C and 230 °C. The cooling cycle shifts the characteristic phase transition ranges towards lower temperatures; i.e., the rhombohedral phase is revealed between temperatures of 230 °C and 170 °C, while the orthorhombic phase appears between 130 °C and 90 °C. During cooling at 90 °C, the coexistence of two phases (RE and OR) was recorded, while, at 60 °C, the OR phase dominated with a small presence of the RE phase. A favorable set of performance parameters predispose the PBZT_*x*Sn material to microelectronic and micromechatronic applications.

## Figures and Tables

**Table 1:** Mass % of compounds in samples calculated for theoretical and experimental investigations (HP).

	PBZT_0Sn		PBZT_2Sn		PBZT_4Sn		PBZT_6Sn		PBZT_8Sn	
	theor.	exper.	theor.	exper.	theor.	exper.	theor.	exper.	theor.	exper.
**TiO_2_**	0.47	0.20	0.46	0.38	0.45	0.32	0.44	0.45	0.43	0.33
**ZrO_2_**	35.16	30.04	34.4	28.74	33.64	28.86	32.89	27.90	32.13	26.79
**SnO_2_**	0	0	0.88	0.88	1.75	1.36	2.62	2.30	3.49	3.38
**BaO**	1.34	1.48	1.34	1.18	1.33	1.12	1.33	0.84	1.33	1.15
**PbO**	63.03	68.27	62.92	68.82	62.83	68.34	62.72	68.50	62.62	68.35

**Table 2:** Parameters of the PBZT_*x*Sn ceramic samples.

Parameter	PBZT_0Sn	PBZT_2Sn	PBZT_4Sn	PBZT_6Sn	PBZT_8Sn
*ρ* (g/cm^3^)	7.34	7.38	7.42	7.60	7.53
*d* (μm)	1.42	1.40	1.87	1.70	1.86
*ε*_r_ ^a,b^	274	275	269	265	269
tan*δ* ^a,b^	0.119	0.123	0.097	0.064	0.031
*T*_m_ (°C) ^b^	228	221	216	212	205
*ε*_m_ at *T*_m_^b^	12,141	11,526	10,850	10,861	8923
tan*δ* at *T*_m_^b^	0.194	0.194	0.151	0.114	0.056
*P*_r_ (μC/cm^2^) ^a^	0.53	0.40	0.50	0.31	0.30
*E*_c_ (kV/mm) ^a^	1.14	0.77	0.89	0.71	0.69
*P*_max_ (μC/cm^2^) ^a^	1.81	1.85	2.08	1.52	1.63

^a^—at room temperature; ^b^—at 1 kHz.

**Table 3:** The structural analysis performed by the Rietveld method for PBZT_6Sn ceramics.

Cycle	Heating								
Phase	Orthorhombic (OR)			Rhombohedral (RE)		Cubic (C)	*R_p_*	*R_wp_*	*R_B_*
*T* (°C)	*a* (Å)	*b* (Å)	*c* (Å)	*a* (Å)	*c* (Å)	*a* (Å)			
30	5.8653(1)	11.7453(2)	8.2177(1)	-	-	-	11.4	9.45	5.21
60	5.8659(1)	11.7450(2)	8.2232(1)	-	-	-	11.0	8.72	3.40
90	5.8665(1)	11.7451(2)	8.2286(1)	-	-	-	11.1	8.86	3.57
130	5.8668(1)	11.7445(2)	8.2358(1)	-	-	-	13.8	11.5	4.91
170	-	-	-	5.8816(2)	14.3585(5)	-	16.2	14.2	3.17
230	-	-	-	-	-	4.1519(1)	12.5	8.92	2.18
	**Cooling**								
170	-	-	-	5.8805(1)	14.3585(1)	-	15.4	13.6	2.93
130	-	-	-	5.8815(2)	14.3503(5)	-	16.8	15.0	3.27
90	5.8659(1)	11.7450(2)	8.2232(1)	5.8779(1)	14.3359(3)	-	15.5	13.5	3.19
60	5.8667(1)	11.7452(2)	8.2242(2)	5.8786(2)	14.3245(6)	-	11.6	9.44	2.13

## Data Availability

Data are contained within the article.

## Supplementary Materials

The following supporting information can be downloaded at: https://www.mdpi.com/article/10.3390/ma17205072/s1, Figure S1: Enlarged fragment of the X-ray diagram for PBZT_*x*Sn materials for the range of 2θ angles (a) 42°–45° and (b) 53°–55°.
