# Supplementary material for: Properties of Sn-Doped PBZT Ferroelectric Ceramics Sintered by Hot-Pressing Method
Source: Materials (Basel). 2024 Oct 17;17(20):5072. doi: 10.3390/ma17205072 (PMC11509812; doi:10.3390/ma17205072)
Supplement: Supplementary file 1 [file materials-17-05072-s001.zip › materials-3219490-supplementary.pdf]

Supplementary Materials

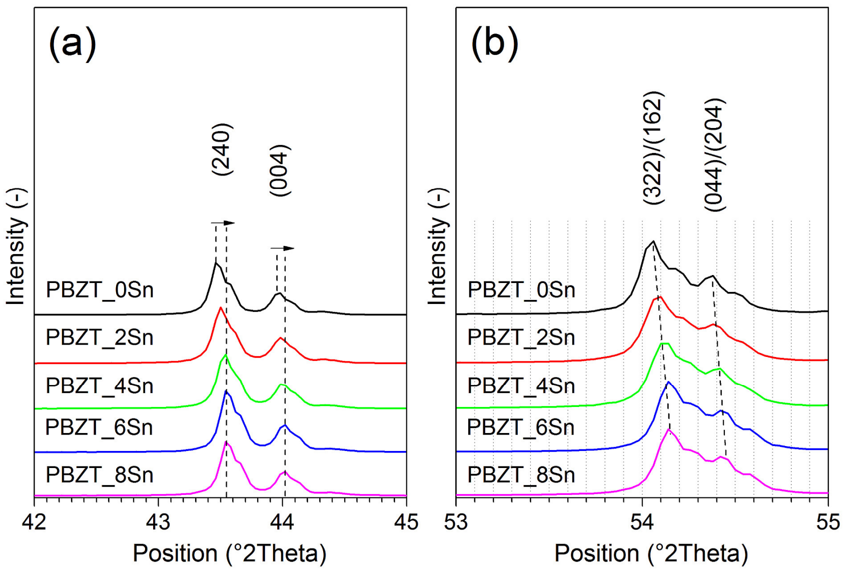

Figure S1. Enlarged fragment of the X-ray diagram for PBZT_*x*Sn materials for the range of 2θ angles (a) 42°–45° and (b) 53°–55°.
